# SimID: Wi-Fi-Based Few-Shot Cross-Domain User Recognition with Identity Similarity Learning

**DOI:** 10.3390/s25165151

**Published:** 2025-08-19

**Authors:** Zhijian Wang, Lei Ouyang, Shi Chen, Han Ding, Ge Wang, Fei Wang

**Affiliations:** 1 School of Software Engineering, Xi’an Jiaotong University, Xi’an 710049, China; wangzhijian@stu.xjtu.edu.cn (Z.W.); ouyanglei_20@stu.xjtu.edu.cn (L.O.); cs2223515409@stu.xjtu.edu.cn (S.C.); 2 State Key Laboratory of Integrated Services Networks, Xidian University, Xi’an 710071, China; 3 School of Computer Science and Technology, Xi’an Jiaotong University, Xi’an 710049, China; dinghan@xjtu.edu.cn (H.D.); gewang@xjtu.edu.cn (G.W.)

**Keywords:** wireless sensor network, Wi-Fi sensing, person identification, transfer learning, few-shot learning, domain adaptation

## Abstract

In recent years, indoor user identification via Wi-Fi signals has emerged as a vibrant research area in smart homes and the Internet of Things, thanks to its privacy preservation, immunity to lighting conditions, and ease of large-scale deployment. Conventional deep-learning classifiers, however, suffer from poor generalization and demand extensive pre-collected data for every new scenario. To overcome these limitations, we introduce SimID, a few-shot Wi-Fi user recognition framework based on identity-similarity learning rather than conventional classification. SimID embeds user-specific signal features into a high-dimensional space, encouraging samples from the same individual to exhibit greater pairwise similarity. Once trained, new users can be recognized simply by comparing their Wi-Fi signal “query” against a small set of stored templates—potentially as few as a single sample—without any additional retraining. This design not only supports few-shot identification of unseen users but also adapts seamlessly to novel movement patterns in unfamiliar environments. On the large-scale XRF55 dataset, SimID achieves average accuracies of 97.53%, 93.37%, 92.38%, and 92.10% in cross-action, cross-person, cross-action-and-person, and cross-person-and-scene few-shot scenarios, respectively. These results demonstrate SimID’s promise for robust, data-efficient indoor identity recognition in smart homes, healthcare, security, and beyond.

## 1. Introduction

**Figure 1:**
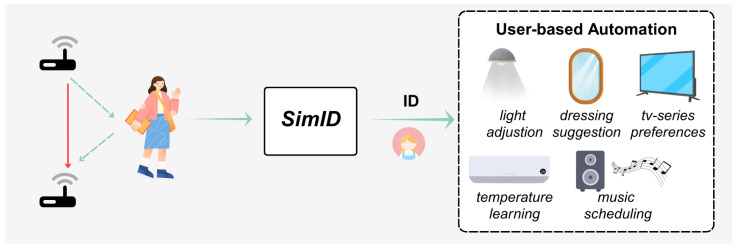
Wi-Fi behavioral biometrics for few-shot user identification.

The rapid advancement of smart home technologies has fueled the demand for highly personalized services that enhance the user experience [1,2]. As shown in Figure 1, through user identification and intelligent automation systems, homes now proactively adapt to individual preferences: lighting adjusts to optimal brightness levels [3]; thermostats regulate indoor temperatures according to users’ behavioral patterns [4] and entertainment systems initiate customized playlists by analyzing historical usage data—all achieved without requiring manual intervention [5,6].

Accurate user identification in smart homes traditionally relies on authentication passwords, fingerprints [7], and facial recognition [8]. While these methods are effective for security, their intrusive nature—requiring deliberate user input or close physical interaction (e.g., entering passwords or scanning fingerprints)—disrupts the seamless flow of daily activities. In contrast, behavior-based identification offers a non-intrusive paradigm by recognizing users through their unique action patterns, such as characteristic movements, habitual gestures, or daily routines. For instance, Wu et al. developed a Kinect-based gesture recognition system using DTW for user identification [9], while NeuralWave demonstrates 87.76% identification accuracy by applying deep learning to Wi-Fi CSI-based gait biometrics [10]. This approach eliminates the need for explicit user cooperation, while still ensuring reliable user identification.

While behavior-based identification shows promise for smart environments, current methods face two limitations in whole-home deployment: (1) constrained operational range [11], and (2) vulnerability to occlusion [12]. To overcome the spatial and occlusion challenges, researchers have turned to ambient Wi-Fi signals. Systems like WiWho [13], FreeSense [14], and WiPIN [15] exploit the fact that human movements and the human body create distinctive perturbations in Wi-Fi propagation patterns. However, their dependence on predefined walking paths or standing positions significantly hinders practical adoption. Alternative approaches using specific daily actions (e.g., microwave operation) [16] demonstrate feasibility but remain constrained to just five common activities. Our investigation reveals XRF55 [17] as the current state-of-the-art in action diversity, supporting 55 distinct motions. Leveraging this comprehensive dataset, we develop a generalized behavior-based framework that achieves robust identification across an unprecedented range of natural behaviors while maintaining high accuracy.

Existing behavior-based identification methods [16] typically require collecting extensive datasets of predefined users and predefined actions to train an identification model, which fundamentally limits the practical applicability since the trained model cannot generalize to new users, unseen actions, or different environments without recollecting new training data and retraining the model—a process that is often labor-intensive and sometimes infeasible in real-world scenarios [13,18,19]. To overcome the limitations, we propose developing adaptive identification systems capable of achieving robust user identification with minimal data collection efforts (e.g., requiring only one or a few samples per user in target settings), which is particularly crucial for enabling practical deployments where exhaustive data gathering is impossible.

Unlike conventional user identification systems, our SimID is to learn high-level identity representations in a feature space. During training, SimID processes Wi-Fi inputs, optimizing the features to maximize similarity between samples from the same identity while maximizing separation between different identities. The trained SimID can effectively cluster identities in the feature space without requiring predefined user identities. During deployment, user identification requires only a small support set (also known as a *gallery*) of reference samples. For each new Wi-Fi sample, SimID computes its similarity score to samples in the support set and assigns the user identity of the most similar one. This approach eliminates the need for large-scale data recollections and model retraining—only a few new reference samples need to be added to the support set. Technically, we implement SimID through a Prototypical architecture [20] with an SE-ResNet-based feature encoder [21], modified to process 2D Wi-Fi signals instead of conventional 3D inputs. We evaluate SimID under four experimental conditions: (1) cross-action (with an accuracy of 97.53%), (2) cross-person (with an accuracy of 93.37%), (3) cross-action-and-person (with an accuracy of 92.38%), and (4) cross-person-and-scene (with an accuracy of 92.10%); the results demonstrate high identification accuracy with just one reference sample, significantly improving generalization ability to new settings, and reducing data collection overhead compared with conventional systems. Our contributions can be highlighted as follows:

- We propose SimID, a Wi-Fi-based user identification system that exhibits strong generalization capabilities in new actions, new users, and new scenes.
- SimID is built on Prototypical Network and SE-ResNet that generates high-level identity features from Wi-Fi signals, maximizing the similarity between the same identity while maximizing separation between different identities.
- We evaluate SimID in var scenarios: cross-action, cross-person, cross-action-and-person, and cross-person-and-scene. Extensive experimental results demonstrate that SimID outperforms some other methods, such as Siamese Networks [22] and Relation Network [23].

In addition, our code is released at https://github.com/FairyStories-wzj/SimID (accessed on 1 July 2025).

## 2. Related  Work

### 2.1. Wi-Fi Action Recognition

Wi-Fi-based human activity recognition (HAR) has gained significant attention due to its cost-effectiveness, ease of deployment, and non-intrusive nature. The primary signals used include Received Signal Strength Indicator (RSSI) and Channel State Information (CSI), with CSI being particularly effective for capturing subtle variations caused by human activities. Recent work in Wi-Fi-based HAR has focused on improving accuracy and robustness through a variety of approaches. E-eyes [24] achieves over 96% accuracy in identifying location-oriented activities using existing Wi-Fi infrastructure. Wi-Vi [25] demonstrates seeing through walls by using MIMO interference nulling to track human movements. WiKey [19] recognizes keystrokes with a 97.5% detection rate by analyzing unique CSI-waveforms generated during typing. Widar3.0 [26] introduces domain-independent body velocity profiles, achieving 82.6–92.4% cross-domain accuracy without retraining. Person-in-Wi-Fi [27] performs body segmentation and pose estimation using only 1D Wi-Fi signals, comparable to image-based solutions. CARM [28] provides theoretical models correlating CSI dynamics with human activities, achieving over 96% accuracy. WiHF [29] enables real-time Wi-Fi-based gesture recognition and user identification, achieving over 96% accuracy through seam carving algorithms and a specially designed deep neural network. Wi-Fi-based HAR has advanced a lot in action recognition [19,24,30,31], temporal action localization [31,32], action summarization [32], and human pose estimation [27,33,34], yet challenges like limited data and environmental sensitivity remain, especially in few-shot scenarios.

### 2.2. Wi-Fi User Identification

In recent years, researchers have researched and developed various methods for Wi-Fi-based user identification. Zhang et al. [18] pioneered the use of Wi-Fi Channel State Information (CSI) for human identification, extracting the feature from the user’s gait. WiWho [13] utilizes users’ gait information for human identification, attaining an accuracy rate ranging from 92% to 80% within groups of 2 to 6 individuals. Korany et al. [35] conducted feature separation for three individuals walking in different directions, created spectrograms, and matched them against a fingerprint dataset, achieving an identification accuracy of 82%. Wang et al. [36] developed a segmentation algorithm to distinguish gait data from breathing data, combined with a weighted subcarrier selection technique to enhance anti-interference performance. Shi et al. [16] analyzed Wi-Fi signal features derived from both walking and other activities, demonstrating that human activities can serve as a reliable means for human identification. Wihi [37] utilized a recurrent neural network model incorporating LSTM blocks to analyze the power and energy distribution of CSI data, enabling human identification. WiAu [38] integrated CNN and ResNet to construct its system model, with CNN reducing the dimensionality of the raw data and ResNet capturing advanced features from the CSI samples. WiPIN [15] provides a user-friendly and robust Wi-Fi-based identification system. BodyPIN [39] proposes a system prototype that leverages Wi-Fi for continuous user authentication. WiDDF-ID [40] successfully adapts DenseNet [41], originally designed for image recognition, to the task of Wi-Fi CSI-based identity recognition. WiAi-ID [42] builds upon CSI feature extraction by incorporating adversarial learning, encouraging the model to focus more on identity-related features while suppressing irrelevant variations. WiDual [43] builds upon a dual-attention mechanism and ResNet [44] architecture, and innovatively integrates identity recognition with action recognition as complementary tasks. This joint learning strategy not only expands the potential application scenarios but also improves overall recognition accuracy.

Existing behavior-based and gait-based user identification approaches require collecting a large number of samples and updating identification models when encountering new individuals in new scenarios, which is not efficient in practice. Thus, we propose SimID to address this issue.

### 2.3. Few-Shot Learning

In classification tasks, Few-shot Learning (FSL) or One-shot Learning (OSL) refers to the ability of a model to correctly distinguish the categories in the query set, even when only a very limited number of samples per category are available in the support set. Conventional deep learning methods typically rely on large-scale labeled datasets for training, whereas FSL aims to address the challenge of effective classification when data acquisition is costly or labeling is difficult. The research on FSL can be traced back to Bayesian One-Shot Learning, proposed by Li et al. [45], which leveraged Bayesian methods to achieve object classification under extremely limited sample conditions. In the field of deep learning, various network architectures have been developed to enable such capabilities. For instance, Siamese Networks, introduced by Koch et al. [22], pair samples and extract their high-dimensional feature representations using two networks with identical parameters. By comparing the distance between these high-dimensional features, Siamese Networks enable One-shot Learning classification. Similarly, Prototypical Networks, proposed by Snell et al. [20], aggregate support set samples of the same category into a unified “prototype” in high-dimensional space and classify query samples based on their distances to these prototypes, demonstrating strong FSL capabilities. Building upon this, Relation Networks, introduced by Sung et al. [23], replace simple distance metrics with a relation module to compute similarity, further enhancing network performance. Other approaches, such as Matching Networks [46], Triplet Networks [47], and Meta-Learning [48], serve as representative examples of effective methodologies for FSL and OSL.

These methods extract high-dimensional representations of samples and classify them based on feature comparisons. In this paper, we apply FSL methods to Wi-Fi-based user identification and propose SimID to enhance the user identification in situations involving new individuals in new scenes.

## 3. SimID System

**Figure 2:**
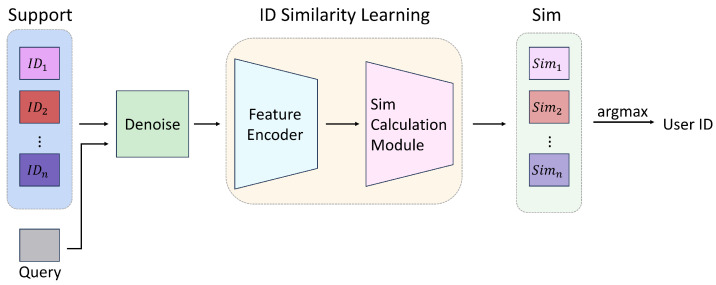
In the user identity verification process, the user’s information is compared with the support data. After undergoing a denoising operation, the similarities are learned through ID similarity learning. The output similarities are then compared to produce the final result of the user ID.

SimID is a user recognition system that leverages variations in Wi-Fi signals caused by human activity. When a user moves within a Wi-Fi environment, the resulting signal fluctuations implicitly encode information about the user’s body shape, movement habits, and gait, which can be used to identify the individual. In this process, the Wi-Fi signals affected by the user’s movements are treated as a *query*, and SimID determines the user’s identity based on this *query*, as shown in Figure 2.

A key advantage of SimID lies in its ability to perform few-shot cross-domain user recognition, meaning it can generalize to new environments, unseen users, and novel actions. Unlike conventional user recognition approaches that rely on training a classifier followed by direct prediction, SimID maintains a small number of identity-matching templates for each user, forming a *support set*. User recognition is then performed by measuring the similarity between the *query* and the templates in the *support set*. Under this design, the training of SimID becomes a similarity learning task, rather than a conventional classification problem. Next, we are going to define the problem setting.

### 3.1. Problem Formulation

Suppose we have a training set $D_{\mathrm{train}}$ with $U_{\mathrm{train}}$ user identities performing $A_{\mathrm{train}}$ categories of actions, and a test set $D_{\mathrm{test}}$ with $U_{\mathrm{test}}$ user identities performing $A_{\mathrm{test}}$ categories of actions to evaluate SimID. Since SimID is designed to generalize to new users and novel user movements that generate unseen queries, the sets of users in training and testing, denoted as $U_{\mathrm{train}}$ and $U_{\mathrm{test}}$, may be overlapping, partially overlapping, or even completely disjointed in terms of user identities. Similarly, the sets of actions $A_{\mathrm{train}}$ and $A_{\mathrm{test}}$ may also be partially or entirely non-overlapping.

Our goal is to maximize SimID’s user recognition accuracy on the test set $D_{\mathrm{test}}$, given the training set $D_{\mathrm{train}}$ and the trained SimID.

### 3.2. Training Strategy

#### 3.2.1. Signal Processing

Raw Channel State Information (CSI) signals inevitably contain noise from hardware imperfections, environmental interference, and multipath effects. This noise can mask the subtle signal variations caused by human movements, making identification tasks challenging.

We employ a second-order Butterworth low-pass filter [49] with a normalized cutoff frequency of 0.02 to denoise raw CSI as in WiPIN [15]. The Butterworth filter was selected for its maximally flat frequency response in the passband, which preserves the integrity of human motion information while effectively removing high-frequency noise components. We apply this filter to each subcarrier independently using a forward-backward filtering technique to ensure zero-phase distortion.

**Figure 3:**
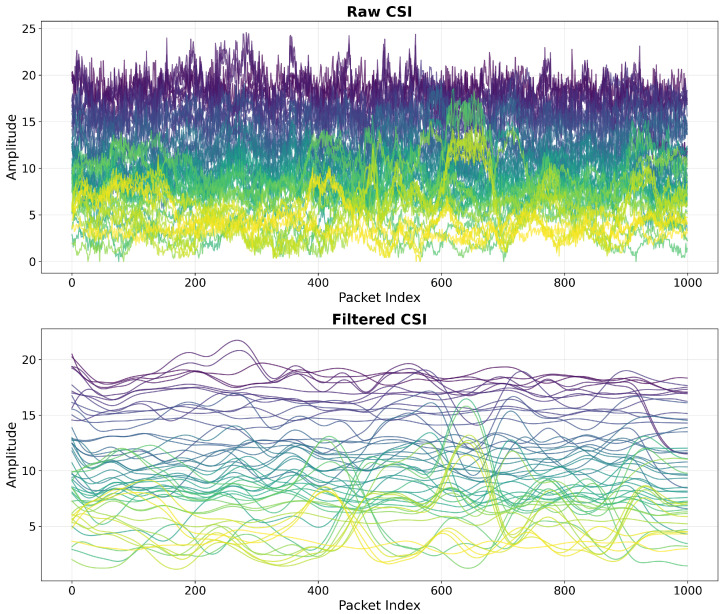
Noise removal using a Butterworth filter (different colors represent different subcarriers).

Figure 3 illustrates the effectiveness of our denoising process through a visual comparison of raw and filtered CSI signals, showing substantially smoother trajectories that better represent the underlying patterns of human movement. The filtered signals maintain essential amplitude variations while eliminating rapid oscillations that typically represent noise rather than meaningful data.

#### 3.2.2. Training Support Set and Training Query Set Sampling

Our training stage consists of a large number of iterations, and in each iteration we first sample a training support set ($D_{\mathrm{support}}^{\mathrm{train}}$) and a training query set ($D_{\mathrm{query}}^{\mathrm{train}}$) from the training set $D_{\mathrm{train}}$. In the sampling process, we first randomly select an action category of $a_{\mathrm{train}}$ from $A_{\mathrm{train}}$, and then sample $n_{\mathrm{train}}\cdot \left|U_{\mathrm{train}}\right|$ data instances that correspond to $a_{\mathrm{train}}$ from $D_{\mathrm{train}}$ to form the training support set $D_{\mathrm{support}}^{\mathrm{train}}{|}_{a_{\mathrm{train}}}$. This means that for each user performing action $a_{\mathrm{train}}$, $n_{\mathrm{train}}$ instances are sampled from the training set $D_{\mathrm{train}}$. The sampling can be noted as Equation (1).

(1) $$D_{\mathrm{support}}^{\mathrm{train}}{|}_{a_{\mathrm{train}}}=\underset{u_{\mathrm{train}}\in U_{\mathrm{train}}}{⋃}X_{u_{\mathrm{train}}},\;\;\mathrm{where}\;X_{u_{\mathrm{train}}}\subset D_{\mathrm{train}}|\left(u_{\mathrm{train}},a_{\mathrm{train}}\right),\;\left|X_{u_{\mathrm{train}}}\right|=n_{\mathrm{train}}$$

where $D_{\mathrm{train}}|\left(u_{\mathrm{train}},a_{\mathrm{train}}\right)$ denotes data instances that correspond to the user $u_{\mathrm{train}}$ performing action $a_{\mathrm{train}}$; $X_{u_{\mathrm{train}}}$ denotes $n_{\mathrm{train}}$ sampled data instances from $D_{\mathrm{train}}|\left(u_{\mathrm{train}},a_{\mathrm{train}}\right)$; the symbol ⋃ denotes a set union operation, meaning that the sampled instances from each user are aggregated into the training support set $D_{\mathrm{support}}^{\mathrm{train}}$ in each training iteration.

Meanwhile, in each training iteration, we also sample *B* data instances from $D_{\mathrm{train}}|a_{\mathrm{train}}$ to form the training query set as in Equation (2).

(2) $$D_{\mathrm{query}}^{\mathrm{train}}{|}_{a_{\mathrm{train}}}=\left\{x_{1},x_{2},\ldots ,x_{B}\;|\;x_{i}\in D_{\mathrm{train}}{\left|a_{\mathrm{train}}\setminus D_{\mathrm{support}}^{\mathrm{train}}\right|}_{a_{\mathrm{train}}},\;1\leq i\leq B\right\}$$

where *B* is a hyperparameter representing the batch size used during training.

#### 3.2.3. Prototype Computation and Loss Function

With the training support set, $D_{\mathrm{support}}^{\mathrm{train}}{|}_{a_{\mathrm{train}}}$, we compute the user identity feature center, also named *prototype*, for each user $u_{\mathrm{train}}\in U_{\mathrm{train}}$ with Equation (3).

(3) $$p_{u_{\mathrm{train}}}^{\mathrm{support}}=\frac{1}{n_{\mathrm{train}}}\sum_{i=1}^{n_{\mathrm{train}}}f\left(x_{u_{\mathrm{train}},i}\right)\;\;\mathrm{where}\;x_{u_{\mathrm{train}},i}\in X_{u_{\mathrm{train}}}$$

where $X_{u_{\mathrm{train}}}$ shares the same meaning in Equation (1); *f* represents the user feature encoder. In SimID, we adapt SE-ResNet [21] following the method in ARIL [30] by replacing its 2D convolutions with 1D convolutions to handle Wi-Fi time-series data. This modification reduces the number of trainable parameters from 4.94 M in the 2D model to just 3.16 M in the 1D version.

For each $x_{i}^{\mathrm{query}}\in D_{\mathrm{query}}^{\mathrm{train}}$, SimID computes its similarity with each prototype in $D_{\mathrm{support}}^{\mathrm{train}}$ and constructs the similarity vector s as in Equation (4).

(4) $$s=[\mathrm{Sim}\left(p_{1}^{\mathrm{support}},f\left(x_{i}^{\mathrm{query}}\right)\right),\mathrm{Sim}\left(p_{2}^{\mathrm{support}},f\left(x_{i}^{\mathrm{query}}\right)\right),\cdots ,\mathrm{Sim}\left(p_{U_{\mathrm{train}}}^{\mathrm{support}},f\left(x_{i}^{\mathrm{query}}\right)\right)]$$

where Sim(·) is to compute feature similarity between the prototype and query, and the computation process is as in Equation (5).

(5) $$\mathrm{Sim}\left(p^{\mathrm{support}},f\left(x^{\mathrm{query}}\right)\right)=\mathrm{BN}2\left(W\cdot \mathrm{BN}1(\Delta \circ \Delta )+b\right)$$

where $\Delta =p^{\mathrm{support}}-f\left(x^{\mathrm{query}}\right)$; BN1, BN2, *W*, and b are two batch normalization layers and the weight and bias of one linear layer, respectively. ∘ means element-wise multiplication, which refers to the operation where the corresponding elements of two vectors are multiplied together.

The user corresponding to the highest similarity score is regarded as the predicted identity. To enable training under this setup, we apply a softmax function to normalize the similarity scores, i.e., s=SoftMax(s), and then use the cross-entropy loss to measure the discrepancy between the predicted identity and the ground truth with Equation (6).

(6) $$L=-\sum_{i=1}^{B}\log\left(s_{u_{i}}\right)$$

where $u_{i}$ represents the ground truth identity of the query instance $x_{i}^{\mathrm{query}}$.

According to this loss function, the higher the similarity score assigned to the correct category (i.e., a higher $s_{u_{i}}$), the lower the resulting loss. At the same time, due to the use of the SoftMax function, the lower the similarity scores assigned to incorrect categories, the smaller the loss. In this way, the loss function guides the model to optimize its parameters such that the similarity between samples of the same identity is maximized.

### 3.3. Test Strategy

The testing protocol mirrors the training procedure. For each iteration, we first sample an action category $a_{\mathrm{test}}$ from the test set $D_{\mathrm{test}}$ and, for each user, sample $n_{\mathrm{test}}$ instances of that action, as defined in Equation (7), to form the test support set $D_{\mathrm{support}}^{\mathrm{test}}$. The value of $n_{\mathrm{test}}$ thus serves as the *n*-shot learning hyperparameter *n*.

(7) $$D_{\mathrm{support}}^{\mathrm{test}}{|}_{a_{\mathrm{test}}}=\underset{u_{\mathrm{test}}\in U_{\mathrm{test}}}{⋃}X_{u_{\mathrm{test}}},\;\;\mathrm{where}\;X_{u_{\mathrm{test}}}\subset D_{\mathrm{test}}|\left(u_{\mathrm{test}},a_{\mathrm{test}}\right),\;\left|X_{u_{\mathrm{test}}}\right|=n_{\mathrm{test}}$$

We further compute each user’s feature mean (the prototypes) as in Equation (8).

(8) $$p_{u_{\mathrm{test}}}^{\mathrm{support}}=\frac{1}{n_{\mathrm{test}}}\sum_{i=1}^{n_{\mathrm{test}}}f\left(x_{u_{\mathrm{test}},i}\right)\;\;\mathrm{where}\;x_{u_{\mathrm{test}},i}\in X_{u_{\mathrm{test}}}$$

We then randomly select one remaining sample of the same action from the test set as the query. By computing similarities between the query and all prototypes, the identity corresponding to the highest-similarity prototype is taken as the predicted user, and the result is recorded. This process is repeated for 5000 iterations, and the proportion of correctly identified queries is reported as the accuracy.

### 3.4. Hyper Parameters

In our implementation, hyperparameters are experimentally set as shown in Table 1.

## 4. Evaluation

### 4.1. Datasets and Data Splitting

We conduct our experiments using the XRF55 dataset [17], which contains comprehensive human activity recognition (HAR) data collected from 31 subjects performing 55 distinct action classes with 20 repetitions each. The dataset covers five categories of human activities: Human-object Interactions, Human-human Interactions, Fitness, Body Motions, and Human-computer Interactions, and it comprises four scenarios in total, as shown in Table 2. XRF55 is a large-scale multimodal dataset, including Wi-Fi Channel State Information (CSI), millimeter-wave radar heatmaps, Radio Frequency Identification tags’ recordings, and videos.

The XRF55 dataset employs a setup in which one ThinkPad X201 laptop (Lenovo, Beijing, China), outfitted with an Intel 5300 wireless card, acts as the Wi-Fi signal transmitter, while three additional laptops serve as receivers. These four devices are symmetrically arranged at the vertices of a 3.1m×3.1m square area, and at a height of 1.2m. Data collection takes place as participants perform predefined actions positioned at the center of this square layout.

The transmitter is configured to emit packets at a frequency of 200 packets per second, utilizing a single antenna and operating in High Throughput mode (IEEE 802.11n [50]) on channel 128 (5.64 GHz). Each of the three receivers listens to the same channel through three antennas, forming a total of nine independent wireless links. A specialized CSI collection tool is installed across all transceivers to facilitate channel estimation, allowing the system to extract Channel State Information (CSI) corresponding to 30 OFDM subcarriers. The resulting CSI is represented as a tensor of dimensions (200t)×1×3×3×30, where *t* denotes the duration of each recording session in seconds. In this dataset, *t* is fixed at 5, meaning that each sample reflects a continuous 5 s action recording.

In this work, we specifically utilize CSI data. In addition, to maintain focus on single-user identification (the current scope SimID), we exclude seven human–human interaction activities from our evaluation as these involve two participants simultaneously. We formally define the user identity set $U:=\{U_{1},\ldots ,U_{31}\}$ containing 31 subjects and the action set $A:=\left\{A_{1},\ldots ,A_{55}\right\}\setminus \left\{A_{16},\ldots ,A_{22}\right\}$ comprising 48 actions after excluding human-human interactions.

We evaluate SimID under the following four distinct data splitting settings, each designed to replicate real-world deployment conditions:

(1) **Cross-Person-Cross-Scene (CPCS):** In a practical setting, we may deploy SimID in new scenes for new users. We use samples from all four scenes to evaluate SimID ’s generalization capabilities. The training phase utilizes actions A from users $U_{\mathrm{train}}=U\setminus \left\{U_{3\ldots 7},U_{13},U_{23},U_{24},U_{31}\right\}$ of Scene 1. To evaluate SimID on unseen subjects from unseen scenes, we define the test user set as $U_{\mathrm{test}}=\left\{U_{3\ldots 7},U_{13},U_{23},U_{24},U_{31}\right\}$ from three other scenes. Additionally, the action set is defined as $A_{\mathrm{train}}=A_{\mathrm{test}}=A.$
(2) **Cross-Action (CA):** In practical deployments, users may perform actions that were never observed during system training. We use samples from Scene 1 to evaluate SimID ’s generalization capabilities. The training phase utilizes actions $A_{\mathrm{train}}=A\setminus \left\{A_{12\ldots 15},A_{27\ldots 30},A_{41\ldots 44},A_{52\ldots 55}\right\}$, where $A_{12\ldots 15}$ are selected from the ‘Human-Object Interaction’ category, $A_{27\ldots 30}$ from ‘Fitness’, $A_{41\ldots 44}$ from ‘Body Motion’, and $A_{52\ldots 55}$ from ‘Human-Computer Interaction’. To evaluate SimID on unseen actions, we define the test set as $A_{\mathrm{test}}=\left\{A_{12\ldots 15},A_{27\ldots 30},A_{41\ldots 44},A_{52\ldots 55}\right\}$. Additionally, the user set is defined as $U_{\mathrm{train}}=U_{\mathrm{test}}=U\setminus U_{31}$ and all the samples are from Scene 1.
(3) **Cross-Person (CP):** In practical deployments, the composition of household users is inherently dynamic—new family members may join or visitors may temporarily interact with the system. We use samples from Scene 1 to evaluate SimID’s generalization capabilities. The training phase utilizes users $U_{\mathrm{train}}=U\setminus \left\{U_{21\ldots 31}\right\}$. To evaluate SimID on unseen subjects, we define the test user set as $U_{\mathrm{test}}=\left\{U_{21\ldots 30}\right\}$. Additionally, the action set is defined as $A_{\mathrm{train}}=A_{\mathrm{test}}=A$ and all samples are from Scene 1.
(4) **Cross-Action-Cross-Person (CACP):** In practical deployments, new household members or visitors may exhibit behavioral patterns that significantly differ from those observed during system training. We use samples from Scene 1 to evaluate SimID’s generalization capabilities. The training phase utilizes actions $A_{\mathrm{train}}=A\setminus \left\{A_{12\ldots 15},A_{27\ldots 30},A_{41\ldots 44},A_{52\ldots 55}\right\}$ from users $U_{\mathrm{train}}=U\setminus \left\{U_{21\ldots 31}\right\}$. To evaluate SimID on unseen subjects’ unseen actions, we have $U_{\mathrm{test}}=\left\{U_{21\ldots 30}\right\}$ and $A_{\mathrm{test}}=\left\{A_{12\ldots 15},A_{27\ldots 30},A_{41\ldots 44},A_{52\ldots 55}\right\}$. All samples are from Scene 1.

**Figure 4:**
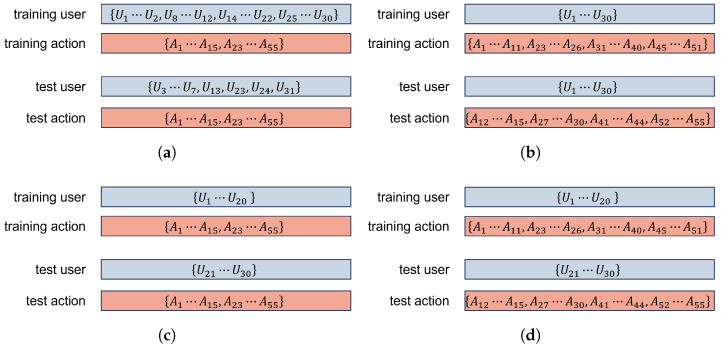
The data splitting settings. (**a**) Cross-Person-Cross-Scene (CPCS); (**b**) Cross-Action (CA); (**c**) Cross-Person (CP); (**d**) Cross-Action-Cross-Person (CACP).

The four kinds of data splitting are illustrated in Figure 4 and the number of training and test set samples is listed in Table 3.

### 4.2. Results

#### 4.2.1. Different Few-Shot Learning Networks

There are various networks that can implement few-shot learning, but their performance varies. To select the best network for our task, we compared three classic few-shot learning networks: Siamese Networks [22], Prototypical Network [20], and Relation Network [23]. We take the same SE-ResNet as the feature encoder of the three networks and evaluate their performance given *n* samples from each user as the support set, i.e., *n*-shot test.

**Figure 5:**
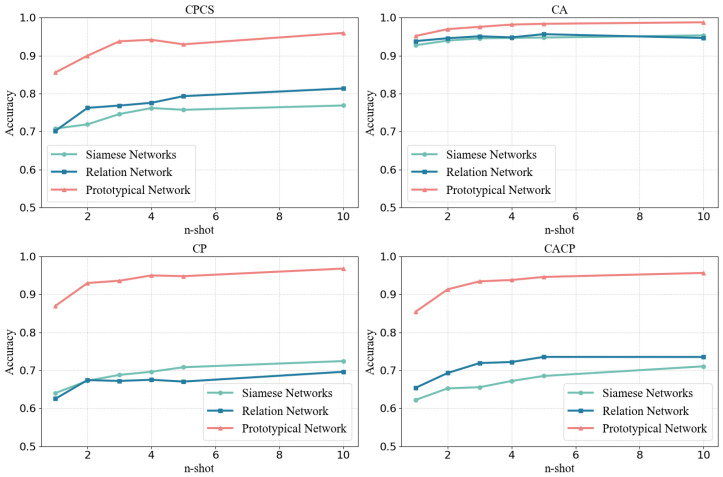
The accuracy on $D_{\mathrm{test}}$ of different networks, different datasets, and different $n_{\mathrm{test}}$, with the same SE-ResNet feature encoder.

The experimental results are shown in Figure 5 and Table 4. It can be observed that Prototypical Network maintained high accuracy in all four data splitting settings, indicating that it possesses few-shot learning capabilities. However, the performance of Siamese Networks and Relation Networks on CPCS, CP, and CACP remains relatively low, indicating their suboptimal effectiveness for this type of task. Therefore, we conclude that the Prototypical Network is the best choice among the three.

This performance discrepancy can be attributed to the underlying learning mechanisms of each model. The Siamese Networks rely on contrastive learning based on positive and negative sample pairs, which does not align well with the multi-class classification nature of our task. As a result, it struggles to form discriminative decision boundaries across a large number of unseen classes. The Relation Network, in contrast, employs a relation module to compute similarities between feature vectors. While this approach has shown success in image-based tasks with rich visual semantics, it tends to overfit when applied to Wi-Fi CSI data, which are lower in dimensionality and lack the structural complexity of images. In contrast, our Sim computation method, which is specifically designed for CSI data, achieves superior performance under the same conditions.

One piece of evidence supporting this explanation lies in the observation that the CPCS setting, which involves both cross-person and cross-scene variations, presents a more challenging identification task than the CP setting. However, the performance of the Siamese and Relation Networks does not degrade; in fact, it slightly improves. This counterintuitive result is likely due to the larger training set in the CPCS setting, which partially mitigates the limitations of the Siamese Network’s training mechanism and alleviates the overfitting tendency of the Relation Network.

It can also be observed that all three networks perform better on the CA setting compared with the other three settings (a trend that also appears in subsequent experiments). This is primarily because the goal of our system is to recognize human identities, and CA is the only data splitting setting in which the identities in the training and test sets remain unchanged. The reduced discrepancy between training and testing distributions makes the identification task in CA inherently easier. Therefore, this observation is consistent with our expectations.

Furthermore, as *n* increases, the model’s accuracy tends to improve, suggesting that with more support samples, the model makes fewer errors. However, this improvement becomes less pronounced at higher *n*. For example, the performance gain from 1-shot to 2-shot is more significant than the gain from 5-shot to 10-shot.

In addition, we evaluated the performance of SimID equipped with Prototypical Network and SE-ResNet10 on each individual user over 5000 iterations with respect to other metrics, including accuracy, precision, recall, and F1-score, and we observed that their patterns are similar to those of the multi-class accuracy. Therefore, for the sake of conciseness, only the accuracy-related results are reported here. Detailed results are provided in Appendix A.

#### 4.2.2. Different Feature Encoders

In addition to an appropriate few-shot learning network, a suitable feature encoder is also crucial. To this end, we selected three image recognition networks: ResNet [44], DenseNet [41], and SE-ResNet [21], and we made modifications to them as outlined in Section 3.2.3. Since CSI contains less information compared with images, to prevent overfitting, we choose relatively shallow networks. Among these, we selected DenseNet121 for DenseNet, while for ResNet and SE-ResNet, we chose a shallower version of ResNet18, namely ResNet10, which contains only one basic residual block for every one of the four stages. We will compare the performance of these different feature encoders when paired with the same Prototypical Network. Additionally, to highlight the disparities in feature encoders’ scale, we compared the differences in terms of trainable parameters and the time spent per prediction corresponding to each of them.

**Figure 6:**
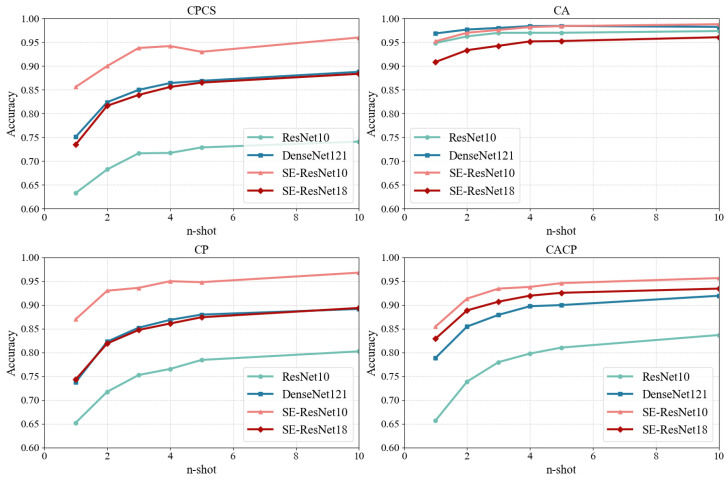
The accuracy on $D_{\mathrm{test}}$ of different feature encoders, different datasets, and different $n_{\mathrm{test}}$, with the same Prototypical Network.

The experimental results are shown in Figure 6, Table 5 and Table 6.

Thanks to the few-shot learning capabilities of the Prototypical Network, all feature encoders achieved high accuracy on all or part of the data splitting settings. However, ResNet10 performs well only on the CA setting, while its performance on the CPCS, CP, and CACP settings is subpar, just like Siamese Networks and Relation Networks. Although DenseNet121 demonstrates relatively stable performance across the four settings, it still slightly underperforms compared with SE-ResNet. Also, as shown in Table 5, its scale and predicting time are much larger, which places it at a disadvantage in terms of training cost and computation efficiency. Among the two SE-ResNet models, SE-ResNet10 outperforms SE-ResNet18, suggesting that a larger model does not necessarily lead to higher accuracy when the network architecture is the same. In conclusion, considering accuracy, stability across data splitting settings, and model scale, SE-ResNet10 is the optimal choice among them.

#### 4.2.3. Different Similarity Computation Methods

To validate the optimization effect of replacing the conventional L2 distance in the Prototypical Network with the Sim calculation module mentioned in Section 3.2.3, we designed an ablation experiment. The experiment compares the performance of two networks—one utilizing L2 distance and the other employing the Sim calculation module—across four data splitting settings, while controlling for variables such as feature encoder, few-shot learning network, and hyper parameters.

**Figure 7:**
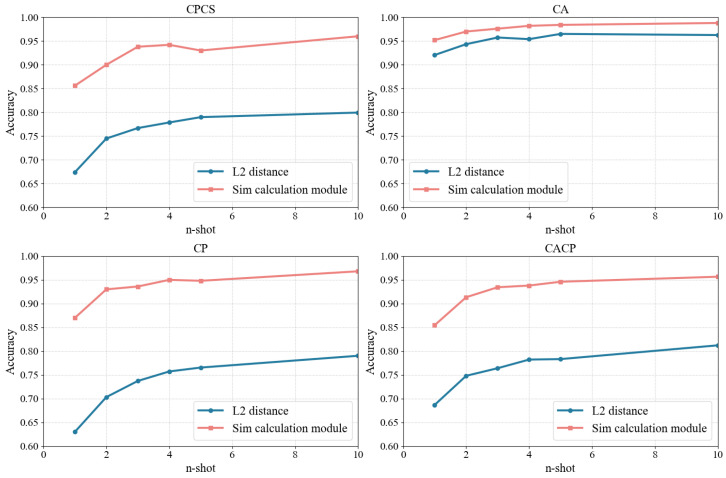
The accuracy on $D_{\mathrm{test}}$ of different similarity computation methods, different data splitting settings, and different $n_{\mathrm{test}}$, with the same Prototypical Network and SE-ResNet feature encoder.

The experimental results are shown in Figure 7 and Table 7. It can be observed that, across all four cross-domain settings, the network with the Sim computation module consistently achieves higher accuracy than the network employing the conventional L2 distance. Moreover, the improvement tends to be more significant when the number of shots is smaller. This suggests that the use of the Sim calculation module is an effective positive optimization, particularly in scenarios with limited data, where the network is more prone to making errors.

#### 4.2.4. SimID vs. Conventional Methods

To evaluate the improvement of SimID over conventional approaches, we select three representative CSI-based Wi-Fi identification systems that adopt conventional methodologies, namely WiDFF-ID [40], WiAi-ID [42], and WiDual [43], and compare their performance with that of SimID. Specifically, WiDFF-ID is a conventional classification system based on DenseNet, while WiAi-ID builds upon CNN-based feature extraction and further employs adversarial learning to encourage the model to focus on inter-person differences. WiDual is a ResNet-based system that incorporates a dual-attention mechanism and jointly learns identity and action recognition as complementary tasks.

Notably, since all the baselines rely on conventional classification methods, they are incapable of identifying identities not seen during training. Therefore, for the CPCS, CP, and CACP settings, we perform a partitioning of the test set. For each user and each action in $D_{\mathrm{test}}$, we randomly select $n_{\mathrm{test}}$ samples to form a *fine-tuning test set*, denoted as $D_{\mathrm{finetune}}^{\mathrm{test}}$. The classifiers of these conventional systems are then fine-tuned on $D_{\mathrm{finetune}}^{\mathrm{test}}$, and their performance is subsequently evaluated on the remaining samples, i.e., $D_{\mathrm{test}}\setminus D_{\mathrm{finetune}}^{\mathrm{test}}$.

**Figure 8:**
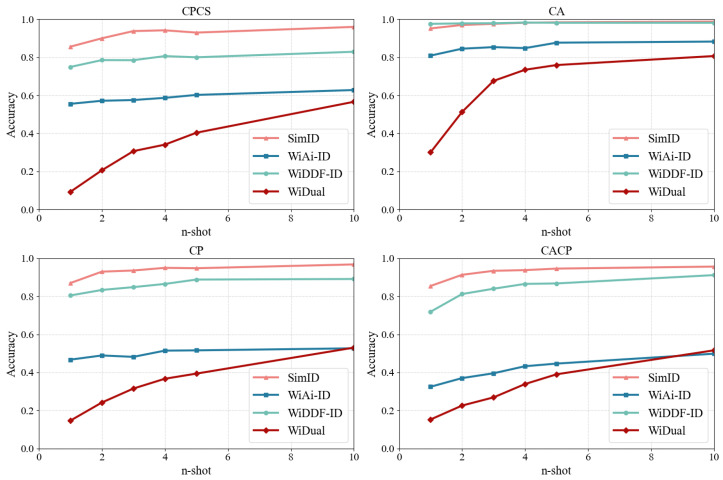
The accuracy of different identification methods, different data splitting settings, and different $n_{\mathrm{test}}$.

The experimental results are presented in Figure 8 and Table 8.

It can be observed that since the CA data splitting setting does not contain any unseen users absent from the training set in the test set, the performance of WiDFF-ID, which is based on conventional classification methods, remains relatively strong. However, in the CPCS, CP, and CACP settings, when the model encounters identities that were not present in the training data, the performance of WiDFF-ID degrades significantly. This indicates that simple fine-tuning of the classifier is insufficient. Achieving high accuracy still requires extensive retraining with a large amount of data. This phenomenon is even more pronounced in the case of WiAi-ID.

Although WiAi-ID employs a domain discriminator that adversarially learns with the classifier to reduce the model’s reliance on non-identity-related features [42], it fails to maintain performance when the person, action, or scene differs between the training and test sets. In such cases, the domain discriminator can no longer accurately distinguish between domains, causing the adversarial learning to break down. Furthermore, unlike WiDFF-ID, which utilizes DenseNet as a feature extractor, WiAi-ID adopts a conventional multi-layer CNN, resulting in weaker feature representation capacity. Coupled with a larger classifier than that of WiDFF-ID, WiAi-ID requires even more data for effective fine-tuning, ultimately leading to inferior performance compared with WiDFF-ID and SimID.

As for WiDual, although it extends the ResNet-based identity recognition framework by incorporating attention mechanisms and joint learning of dual tasks, its performance on the dataset remains poor. One possible reason is the significant distribution shift between the training and testing sets, which may cause the model to focus attention on relevant regions during training but fail to do so during testing, sometimes even underperforming compared with WiDDF-ID, which does not utilize any attention mechanism. Another possible explanation is that WiDual contains two classification heads due to its dual-task design, and the extremely limited number of samples in $D_{\mathrm{finetune}}^{\mathrm{test}}$ is insufficient for effective fine-tuning of both branches simultaneously.

Compared with other conventional methods, SimID not only eliminates the need for fine-tuning but also demonstrates superior generalization in cross-domain identification tasks through its effective few-shot learning capability. These results highlight its promising potential for broad real-world applications.

## 5. Discussion

In indoor identity recognition tasks, the *working distance* is a critical characteristic of a recognition system, as it determines both the placement of sensing devices and the spatial extent in which users can perform activities. For SimID, since all related experiments are conducted on the XRF55 dataset—where Wi-Fi antennas are positioned at the four corners of a 3.1m×3.1m square [17]—we consider the effective working distance of SimID to be confined within this area.

To provide a more comprehensive understanding of SimID’s application potential, we compare its working distance with those of other representative methods mentioned in Section 4.2.4. The comparative results are summarized in Table 9.

As shown in Table 9, although there are some differences among the systems, the working distance of SimID remains within the same order of magnitude as those of existing systems. This indicates that SimID possesses a comparable capability for indoor deployment.

It is worth noting that while the working distance used in our experiments is 3.1m×3.1m, this does not imply that SimID is restricted to operating solely within this range. Rather, increasing the working distance typically leads to a larger gap between the transmitter and receivers, which may introduce additional identity-irrelevant interference into the CSI signals, thereby degrading recognition performance. However, as demonstrated in Section 4.2.4, under the same working distance, SimID achieves higher accuracy than conventional methods. This suggests that SimID has the potential to maintain similar accuracy to conventional approaches even under larger working distances.

## 6. Conclusions

In this paper, we have introduced SimID, a few-shot Wi-Fi user recognition framework that abandons conventional classification in favor of learning identity similarity in feature space. By mapping user signal patterns into a high-dimensional manifold and enforcing greater similarity for samples from the same individual, SimID allows new users to be enrolled with as few as one template sample—no retraining required. Its design naturally generalizes to unseen movement patterns and unfamiliar environments, delivering robust cross-domain performance.

Our extensive evaluation on a large-scale dataset confirms SimID’s data efficiency and generalization capabilities. We achieve average accuracies of 97.53%, 93.37%, 92.38%, and 92.10% in the cross-action, cross-person, cross-action-and-person, and cross-person-and-scene few-shot scenarios, respectively. These results demonstrate that SimID can serve as a practical, privacy-preserving solution for indoor identity recognition—enabling truly “set-and-forget” smart home systems and personalized IoT services without burdening users with extensive data collection.

While SimID excels in few-shot settings, it does not yet address zero-shot recognition of entirely unseen identities. As envisioned by a Wi-Fi sensing generalizability survey [51], in future work, we will investigate zero-shot and adaptive template selection strategies to further enhance SimID’s resilience and accuracy across even more challenging deployment scenarios.

## Figures and Tables

**Table 1:** Hyperparameters in Practice.

Hyper Parameter	Meaning	Value
*B*	size of the training query set, or batch size	128
$n_{\mathrm{train}}$	number of samples per category in the training support set	4
$n_{\mathrm{test}}$	number of samples per category in the test support set, or shot	1,2,3,4,5,10
maxiter	the total number of iterations executed during training	20,000
lr	learning rate	0.1 *

* However, for the CACP data splitting setting (as referenced in Section 4.1), the initial learning rate is set to 0.1 and decays exponentially by a factor of 0.8 every 100 iterations, until it reaches a minimum threshold of $10^{-6}$.

**Table 2:** The XRF55 [17] dataset comprises four distinct experimental scenes with varying participant compositions: Scene 1 includes 30 subjects (Subjects 1–30), Scene 2 includes 3 subjects (Subjects 5, 24, 31), Scene 3 includes 3 subjects (Subjects 6, 7, 23), and Scene 4 includes 3 subjects (Subjects 3, 4, 13).

Scene	Scene 1	Scene 2	Scene 3	Scene 4
Identity	$\left\{U_{1},U_{2},\ldots ,U_{30}\right\}$	$\left\{U_{5},U_{24},U_{31}\right\}$	$\left\{U_{6},U_{7},U_{23}\right\}$	$\left\{U_{3},U_{4},U_{13}\right\}$

**Table 3:** The number of data samples across four different data splitting settings.

	CPCS	CA	CP	CACP
train	21,120	19,200	19,200	12,800
test	8640	9600	9600	3200
Total	29,760	28,800	28,800	16,000

**Table 4:** The accuracy of $D_{\mathrm{test}}$ in different networks, different datasets, and different $n_{\mathrm{test}}$, with the same SE-ResNet feature encoder.

Network	Dataset	*n*-Shot in Test						
		1	2	3	4	5	10	Average
Siamese Networks	CPCS	70.83%	71.93%	74.65%	76.22%	75.76%	76.90%	74.38%
	CA	92.76%	93.98%	94.56%	94.70%	94.78%	95.36%	94.36%
	CP	64.03%	67.27%	68.81%	69.65%	70.83%	72.45%	68.84%
	CACP	62.23%	65.27%	65.57%	67.21%	68.53%	71.07%	66.65%
	average	72.46%	74.61%	75.90%	76.95%	77.48%	78.95%	76.06%
Relation Network	CPCS	70.23%	76.27%	76.88%	77.60%	79.35%	81.38%	76.95%
	CA	93.85%	94.60%	95.12%	94.83%	95.67%	94.67%	94.79%
	CP	62.55%	67.45%	67.22%	67.53%	67.05%	69.63%	66.90%
	CACP	65.42%	69.32%	71.93%	72.20%	73.55%	73.53%	70.99%
	average	73.01%	76.91%	77.79%	78.04%	78.91%	79.80%	77.41%
Prototypical Network	CPCS	85.60%	90.00%	93.80%	94.20%	93.00%	96.00%	**92.10%**
	CA	95.20%	97.00%	97.60%	98.20%	98.40%	98.80%	**97.53%**
	CP	87.00%	93.00%	93.60%	95.00%	94.80%	96.80%	**93.37%**
	CACP	85.46%	91.34%	93.44%	93.80%	94.60%	95.66%	**92.38%**
	average	88.32%	92.84%	94.61%	95.30%	95.20%	96.82%	**93.85%**

**Table 5:** The size and computational time of different feature encoders with the same Prototypical Network. Each encoder is evaluated on the CPCS data splitting setting with 1-shot learning and 500 runs. Reported inference time (mean ± standard deviation) includes feature extraction and similarity computation, excluding signal processing and data loading. All experiments are conducted on a personal computer equipped with an NVIDIA RTX 3080 Ti GPU and an 11th Gen Intel Core i7 CPU.

Feature Encoder	ResNet10	DenseNet121	SE-ResNet18	SE-ResNet10
Trainable parameters	3.11 M	5.64 M	5.37 M	3.16 M
Inference time (ms)	5.69±0.027	26.79±0.016	7.47±0.013	4.81±0.014
Accuracy	79.62%	88.39%	87.87%	93.85%

**Table 6:** The accuracy on $D_{\mathrm{test}}$ of different feature encoders, different datasets, and different $n_{\mathrm{test}}$, with the same Prototypical Network.

Feature Encoder	Dataset	*n*-Shot in Test						
		1	2	3	4	5	10	Average
ResNet10	CPCS	63.28%	68.24%	71.64%	71.72%	72.88%	74.10%	70.31%
	CA	94.84%	96.24%	96.98%	97.00%	97.00%	97.36%	96.57%
	CP	65.22%	71.74%	75.26%	76.54%	78.44%	80.24%	74.57%
	CACP	65.70%	73.88%	77.98%	79.78%	81.04%	83.68%	77.01%
	average	72.26%	77.53%	80.47%	81.26%	82.34%	83.85%	79.62%
DenseNet121	CPCS	75.10%	82.40%	84.98%	86.42%	86.90%	88.78%	84.10%
	CA	96.86%	97.68%	98.02%	98.40%	98.44%	98.24%	**97.94%**
	CP	73.74%	82.28%	85.22%	86.86%	87.98%	89.16%	84.21%
	CACP	78.88%	85.46%	87.92%	89.74%	89.98%	91.94%	87.32%
	average	81.15%	86.96%	89.04%	90.36%	90.83%	92.03%	88.39%
SE-ResNet18	CPCS	73.46%	81.64%	83.92%	85.60%	86.52%	88.36%	83.25%
	CA	90.86%	93.34%	94.26%	95.18%	95.26%	96.06%	94.16%
	CP	74.32%	81.90%	84.74%	86.10%	87.42%	89.38%	83.98%
	CACP	82.92%	88.86%	90.70%	91.96%	92.56%	93.44%	90.07%
	average	80.39%	86.44%	88.41%	89.71%	90.44%	91.81%	87.87%
SE-ResNet10	CPCS	85.60%	90.00%	93.80%	94.20%	93.00%	96.00%	**92.10%**
	CA	95.20%	97.00%	97.60%	98.20%	98.40%	98.80%	97.53%
	CP	87.00%	93.00%	93.60%	95.00%	94.80%	96.80%	**93.37%**
	CACP	85.46%	91.34%	93.44%	93.80%	94.60%	95.66%	**92.38%**
	average	88.32%	92.84%	94.61%	95.30%	95.20%	96.82%	**93.85%**

**Table 7:** The accuracy of $D_{\mathrm{test}}$ in different similarity computation methods, different data splitting settings, and different $n_{\mathrm{test}}$, with the same Prototypical Network and SE-ResNet feature encoder.

Distance	Dataset	*n*-Shot in Test						
		1	2	3	4	5	10	Average
L2	CPCS	67.38%	74.48%	76.68%	77.86%	78.98%	79.94%	75.89%
	CA	92.06%	94.34%	95.74%	95.40%	96.50%	96.26%	95.05%
	CP	63.00%	70.32%	73.72%	75.72%	76.54%	79.02%	73.05%
	CACP	68.68%	74.80%	76.40%	78.22%	78.32%	81.22%	76.27%
	average	72.78%	78.49%	80.64%	81.80%	82.59%	84.11%	80.07%
Sim Computation Module	CPCS	85.60%	90.00%	93.80%	94.20%	93.00%	96.00%	**92.10%**
	CA	95.20%	97.00%	97.60%	98.20%	98.40%	98.80%	**97.53%**
	CP	87.00%	93.00%	93.60%	95.00%	94.80%	96.80%	**93.37%**
	CACP	85.46%	91.34%	93.44%	93.80%	94.60%	95.66%	**92.38%**
	average	88.32%	92.84%	94.61%	95.30%	95.20%	96.82%	**93.85%**

**Table 8:** The accuracy of different identification methods, different data splitting settings, and different $n_{\mathrm{test}}$.

Distance	Dataset	*n*-Shot in Test						
		1	2	3	4	5	10	Average
WiDDF-ID	CPCS	74.94%	78.54%	78.50%	80.64%	80.02%	82.92%	79.26%
	CA	97.60%	97.84%	97.92%	98.28%	98.16%	98.16%	**97.99%**
	CP	80.48%	83.38%	84.86%	86.54%	88.84%	89.14%	85.54%
	CACP	71.92%	81.24%	84.02%	86.56%	86.80%	91.18%	83.62%
	average	81.24%	85.25%	86.33%	88.01%	88.46%	90.35%	86.60%
WiAi-ID	CPCS	55.54%	57.16%	57.54%	58.70%	60.24%	62.80%	58.66%
	CA	80.90%	84.54%	85.36%	84.84%	87.70%	88.28%	85.27%
	CP	46.74%	48.94%	48.20%	51.48%	51.62%	52.70%	49.95%
	CACP	32.48%	37.04%	39.58%	43.28%	44.66%	49.86%	41.15%
	average	53.92%	56.92%	57.67%	59.58%	61.06%	63.41%	58.76%
WiDual	CPCS	9.28%	20.62%	30.68%	34.10%	40.36%	56.62%	31.94%
	CA	30.04%	51.28%	67.62%	73.46%	75.94%	80.68%	63.17%
	CP	14.68%	24.20%	31.54%	36.70%	39.40%	53.06%	33.26%
	CACP	15.28%	22.60%	26.90%	33.92%	39.04%	51.64%	31.56%
	average	17.32%	29.68%	39.19%	44.55%	48.69%	60.50%	39.99%
SimID	CPCS	85.60%	90.00%	93.80%	94.20%	93.00%	96.00%	**92.10%**
	CA	95.20%	97.00%	97.60%	98.20%	98.40%	98.80%	97.53%
	CP	87.00%	93.00%	93.60%	95.00%	94.80%	96.80%	**93.37%**
	CACP	85.46%	91.34%	93.44%	93.80%	94.60%	95.66%	**92.38%**
	average	88.32%	92.84%	94.61%	95.30%	95.20%	96.82%	**93.85%**

**Table 9:** The working distances of different Wi-Fi-based identification systems.

System	WiDDF-ID	WiAi-ID	WiDual	SimID
Working distance	1.14m	2.5m×7m	2m×2m	3.1m×3.1m

## Data Availability

No new data were created or analyzed in this study.

## Appendix A

To further examine the performance of SimID in greater detail, we additionally evaluated its Precision, Recall, and F1-Score for each individual user. The experimental results are presented in the following tables. Based on these results, we observed patterns similar to those of the multi-class accuracy. Therefore, for the sake of conciseness, only the accuracy-related results are reported in the main text.

We note that the results reported in the Appendix exhibit slight differences from those presented in the main text. This discrepancy arises because the random seed was not fixed during testing, resulting in variations in the random initialization of certain operations (e.g., data shuffling, sampling, or model inference order). Such stochasticity can lead to minor fluctuations in the evaluation metrics, which is a common phenomenon in experimental studies. Nevertheless, the differences observed are small and do not affect the overall conclusions drawn from the experiments.

Moreover, in multi-class classification, the per-class accuracy—when defined as the ratio of correctly predicted samples of a class to the total number of samples belonging to that class—is mathematically equivalent to the class-specific recall. Therefore, in the tables below, for the same data splitting setting, the accuracy table and the recall table are identical.

**Table A1:** The **accuracy** of SimID on each individual user in data splitting setting **CPCS**.

User	1-Shot	2-Shot	3-Shot	4-Shot	5-Shot	10-Shot	Average
03	85.90%	91.15%	90.86%	94.95%	93.43%	94.65%	91.82%
04	81.24%	87.71%	91.51%	91.46%	91.79%	93.37%	89.51%
05	91.41%	96.04%	97.39%	97.50%	95.70%	98.12%	96.03%
06	77.70%	84.31%	86.95%	85.97%	88.43%	89.63%	85.50%
07	78.53%	84.84%	86.91%	90.12%	89.93%	90.93%	86.88%
13	84.11%	90.30%	91.32%	90.89%	92.99%	94.01%	90.61%
23	79.85%	86.52%	86.00%	86.91%	87.95%	86.80%	85.67%
24	90.47%	94.27%	93.67%	94.54%	95.30%	96.13%	94.06%
31	88.76%	91.27%	92.74%	95.79%	94.24%	95.58%	93.06%
average	84.22%	89.60%	90.82%	92.01%	92.20%	93.25%	90.35%

**Table A2:** The **precision** of SimID on each individual user in data splitting setting **CPCS**.

User	1-Shot	2-Shot	3-Shot	4-Shot	5-Shot	10-Shot	Average
03	81.99%	88.32%	90.02%	88.72%	91.92%	93.65%	89.10%
04	82.37%	88.72%	92.77%	93.58%	92.32%	92.72%	90.41%
05	96.11%	97.21%	98.19%	98.55%	98.24%	99.24%	97.92%
06	77.56%	87.59%	84.83%	88.41%	89.71%	87.83%	85.99%
07	82.91%	87.10%	89.09%	91.71%	91.20%	92.90%	89.15%
13	84.43%	91.59%	91.49%	95.14%	93.70%	95.70%	92.01%
23	74.87%	81.33%	84.91%	83.42%	85.76%	86.80%	82.85%
24	89.13%	91.88%	91.39%	94.71%	93.94%	95.07%	92.69%
31	88.93%	93.31%	94.45%	94.63%	93.20%	95.74%	93.38%
average	84.26%	89.67%	90.79%	92.10%	92.22%	93.30%	90.39%

**Table A3:** The **recall** of SimID on each individual user in data splitting setting **CPCS**.

User	1-Shot	2-Shot	3-Shot	4-Shot	5-Shot	10-Shot	Average
03	85.90%	91.15%	90.86%	94.95%	93.43%	94.65%	91.82%
04	81.24%	87.71%	91.51%	91.46%	91.79%	93.37%	89.51%
05	91.41%	96.04%	97.39%	97.50%	95.70%	98.12%	96.03%
06	77.70%	84.31%	86.95%	85.97%	88.43%	89.63%	85.50%
07	78.53%	84.84%	86.91%	90.12%	89.93%	90.93%	86.88%
13	84.11%	90.30%	91.32%	90.89%	92.99%	94.01%	90.61%
23	79.85%	86.52%	86.00%	86.91%	87.95%	86.80%	85.67%
24	90.47%	94.27%	93.67%	94.54%	95.30%	96.13%	94.06%
31	88.76%	91.27%	92.74%	95.79%	94.24%	95.58%	93.06%
average	84.22%	89.60%	90.82%	92.01%	92.20%	93.25%	90.35%

**Table A4:** The **F1-score** of SimID on each individual user in data splitting setting **CPCS**.

User	1-Shot	2-Shot	3-Shot	4-Shot	5-Shot	10-Shot	Average
03	83.90%	89.71%	90.44%	91.73%	92.67%	94.15%	90.43%
04	81.80%	88.21%	92.14%	92.51%	92.06%	93.04%	89.96%
05	93.70%	96.62%	97.79%	98.02%	96.95%	98.68%	96.96%
06	77.63%	85.92%	85.88%	87.17%	89.07%	88.72%	85.73%
07	80.66%	85.96%	87.99%	90.91%	90.56%	91.90%	88.00%
13	84.27%	90.94%	91.40%	92.97%	93.35%	94.85%	91.30%
23	77.28%	83.85%	85.45%	85.13%	86.84%	86.80%	84.23%
24	89.80%	93.06%	92.51%	94.62%	94.61%	95.60%	93.37%
31	88.85%	92.28%	93.59%	95.20%	93.72%	95.66%	93.22%
average	84.21%	89.62%	90.80%	92.03%	92.20%	93.27%	90.35%

**Table A5:** The **accuracy** of SimID on each individual user in data splitting setting **CA**.

User	1-Shot	2-Shot	3-Shot	4-Shot	5-Shot	10-Shot	Average
01	91.02%	90.70%	93.04%	94.56%	95.57%	94.48%	93.23%
02	96.76%	96.94%	96.71%	98.87%	97.37%	97.01%	97.28%
03	92.74%	93.96%	92.94%	94.05%	95.51%	97.65%	94.47%
04	98.24%	99.40%	99.47%	99.47%	100.00%	99.39%	99.33%
05	90.14%	95.76%	99.37%	95.40%	98.84%	98.15%	96.28%
06	91.14%	94.61%	97.63%	97.55%	97.21%	97.55%	95.95%
07	98.78%	99.33%	99.31%	96.91%	99.36%	98.83%	98.76%
08	96.25%	100.00%	99.44%	100.00%	99.38%	99.38%	99.07%
09	98.19%	97.99%	98.73%	98.82%	100.00%	100.00%	98.95%
10	95.71%	96.36%	98.91%	97.81%	98.77%	99.39%	97.83%
11	99.40%	99.43%	100.00%	100.00%	100.00%	100.00%	99.81%
12	99.36%	99.41%	99.42%	100.00%	100.00%	98.92%	99.52%
13	94.34%	95.71%	99.44%	98.73%	97.35%	97.77%	97.22%
14	83.72%	89.87%	91.95%	90.86%	93.37%	93.42%	90.53%
15	90.06%	92.36%	97.37%	92.77%	94.54%	96.41%	93.92%
16	99.39%	100.00%	100.00%	100.00%	100.00%	100.00%	99.90%
17	95.27%	96.91%	96.95%	100.00%	99.47%	99.45%	98.01%
18	100.00%	99.43%	100.00%	100.00%	100.00%	100.00%	99.90%
19	100.00%	99.41%	100.00%	100.00%	100.00%	100.00%	99.90%
20	95.73%	97.45%	99.37%	98.11%	97.53%	98.97%	97.86%
21	78.98%	94.12%	90.36%	92.90%	92.98%	93.45%	90.47%
22	93.79%	97.08%	94.89%	93.79%	97.31%	99.35%	96.03%
23	93.17%	92.35%	94.59%	92.78%	90.85%	96.43%	93.36%
24	82.39%	91.93%	88.69%	95.63%	94.61%	90.91%	90.69%
25	89.88%	96.59%	95.48%	95.57%	98.73%	98.01%	95.71%
26	91.56%	96.89%	100.00%	98.80%	97.63%	97.58%	97.08%
27	95.32%	93.98%	96.49%	97.02%	95.57%	98.69%	96.18%
28	84.15%	88.30%	91.52%	94.64%	93.90%	94.74%	91.21%
29	100.00%	100.00%	100.00%	100.00%	100.00%	100.00%	100.00%
30	96.51%	98.78%	98.88%	97.01%	98.31%	98.73%	98.04%
average	93.73%	96.17%	97.03%	97.07%	97.47%	97.82%	96.55%

**Table A6:** The **precision** of SimID on each individual user in data splitting setting **CA**.

User	1-Shot	2-Shot	3-Shot	4-Shot	5-Shot	10-Shot	Average
01	93.83%	96.89%	93.63%	92.67%	94.97%	96.25%	94.71%
02	94.71%	95.96%	95.45%	99.43%	98.67%	99.39%	97.27%
03	92.22%	88.61%	94.05%	94.61%	95.51%	93.26%	93.04%
04	98.82%	99.40%	100.00%	98.95%	99.44%	100.00%	99.43%
05	93.43%	96.34%	98.74%	97.08%	98.27%	99.38%	97.21%
06	94.74%	98.14%	99.40%	97.55%	99.43%	99.38%	98.10%
07	97.01%	94.90%	97.30%	98.74%	97.50%	97.69%	97.19%
08	94.48%	97.31%	98.32%	97.97%	100.00%	98.76%	97.81%
09	97.60%	100.00%	99.36%	100.00%	100.00%	99.42%	99.40%
10	92.86%	96.95%	99.45%	96.24%	98.77%	98.19%	97.08%
11	98.82%	99.43%	98.77%	100.00%	99.40%	99.47%	99.31%
12	99.36%	100.00%	100.00%	100.00%	100.00%	100.00%	99.89%
13	99.34%	100.00%	100.00%	100.00%	100.00%	100.00%	99.89%
14	87.27%	91.61%	96.48%	92.44%	93.89%	94.67%	92.73%
15	83.16%	86.83%	92.50%	90.06%	92.02%	93.06%	89.61%
16	100.00%	100.00%	100.00%	100.00%	100.00%	100.00%	100.00%
17	99.38%	100.00%	100.00%	100.00%	100.00%	100.00%	99.90%
18	98.29%	99.43%	99.42%	100.00%	100.00%	100.00%	99.52%
19	96.72%	97.13%	98.18%	98.10%	97.50%	100.00%	97.94%
20	90.75%	97.45%	96.32%	96.89%	96.34%	97.46%	95.87%
21	90.85%	96.39%	93.75%	94.74%	95.78%	96.91%	94.74%
22	84.83%	95.95%	92.78%	95.57%	95.26%	96.20%	93.43%
23	85.71%	91.41%	91.62%	94.89%	94.85%	91.22%	91.62%
24	88.51%	88.10%	91.98%	90.00%	90.29%	96.39%	90.88%
25	93.79%	97.14%	96.57%	96.79%	96.89%	98.67%	96.64%
26	92.76%	95.12%	98.62%	97.62%	98.21%	98.77%	96.85%
27	91.06%	92.31%	95.38%	97.60%	96.79%	95.57%	94.79%
28	88.00%	93.79%	93.79%	94.64%	96.25%	96.43%	93.82%
29	99.37%	100.00%	100.00%	100.00%	100.00%	100.00%	99.89%
30	96.51%	99.39%	98.88%	99.39%	99.43%	97.48%	98.51%
average	93.81%	96.20%	97.02%	97.07%	97.52%	97.80%	96.57%

**Table A7:** The **recall** of SimID on each individual user in data splitting setting **CA**.

User	1-Shot	2-Shot	3-Shot	4-Shot	5-Shot	10-Shot	Average
01	91.02%	90.70%	93.04%	94.56%	95.57%	94.48%	93.23%
02	96.76%	96.94%	96.71%	98.87%	97.37%	97.01%	97.28%
03	92.74%	93.96%	92.94%	94.05%	95.51%	97.65%	94.47%
04	98.24%	99.40%	99.47%	99.47%	100.00%	99.39%	99.33%
05	90.14%	95.76%	99.37%	95.40%	98.84%	98.15%	96.28%
06	91.14%	94.61%	97.63%	97.55%	97.21%	97.55%	95.95%
07	98.78%	99.33%	99.31%	96.91%	99.36%	98.83%	98.76%
08	96.25%	100.00%	99.44%	100.00%	99.38%	99.38%	99.07%
09	98.19%	97.99%	98.73%	98.82%	100.00%	100.00%	98.95%
10	95.71%	96.36%	98.91%	97.81%	98.77%	99.39%	97.83%
11	99.40%	99.43%	100.00%	100.00%	100.00%	100.00%	99.81%
12	99.36%	99.41%	99.42%	100.00%	100.00%	98.92%	99.52%
13	94.34%	95.71%	99.44%	98.73%	97.35%	97.77%	97.22%
14	83.72%	89.87%	91.95%	90.86%	93.37%	93.42%	90.53%
15	90.06%	92.36%	97.37%	92.77%	94.54%	96.41%	93.92%
16	99.39%	100.00%	100.00%	100.00%	100.00%	100.00%	99.90%
17	95.27%	96.91%	96.95%	100.00%	99.47%	99.45%	98.01%
18	100.00%	99.43%	100.00%	100.00%	100.00%	100.00%	99.90%
19	100.00%	99.41%	100.00%	100.00%	100.00%	100.00%	99.90%
20	95.73%	97.45%	99.37%	98.11%	97.53%	98.97%	97.86%
21	78.98%	94.12%	90.36%	92.90%	92.98%	93.45%	90.47%
22	93.79%	97.08%	94.89%	93.79%	97.31%	99.35%	96.03%
23	93.17%	92.35%	94.59%	92.78%	90.85%	96.43%	93.36%
24	82.39%	91.93%	88.69%	95.63%	94.61%	90.91%	90.69%
25	89.88%	96.59%	95.48%	95.57%	98.73%	98.01%	95.71%
26	91.56%	96.89%	100.00%	98.80%	97.63%	97.58%	97.08%
27	95.32%	93.98%	96.49%	97.02%	95.57%	98.69%	96.18%
28	84.15%	88.30%	91.52%	94.64%	93.90%	94.74%	91.21%
29	100.00%	100.00%	100.00%	100.00%	100.00%	100.00%	100.00%
30	96.51%	98.78%	98.88%	97.01%	98.31%	98.73%	98.04%
average	93.73%	96.17%	97.03%	97.07%	97.47%	97.82%	96.55%

**Table A8:** The **F1-score** of SimID on each individual user in data splitting setting **CA**.

User	1-Shot	2-Shot	3-Shot	4-Shot	5-Shot	10-Shot	Average
01	92.40%	93.69%	93.33%	93.60%	95.27%	95.36%	93.94%
02	95.72%	96.45%	96.08%	99.15%	98.01%	98.18%	97.27%
03	92.48%	91.21%	93.49%	94.33%	95.51%	95.40%	93.74%
04	98.53%	99.40%	99.73%	99.21%	99.72%	99.69%	99.38%
05	91.76%	96.05%	99.05%	96.23%	98.55%	98.76%	96.73%
06	92.90%	96.34%	98.51%	97.55%	98.31%	98.45%	97.01%
07	97.89%	97.07%	98.29%	97.82%	98.42%	98.26%	97.96%
08	95.36%	98.64%	98.88%	98.98%	99.69%	99.07%	98.43%
09	97.90%	98.98%	99.04%	99.40%	100.00%	99.71%	99.17%
10	94.26%	96.66%	99.18%	97.02%	98.77%	98.79%	97.45%
11	99.11%	99.43%	99.38%	100.00%	99.70%	99.73%	99.56%
12	99.36%	99.71%	99.71%	100.00%	100.00%	99.46%	99.71%
13	96.77%	97.81%	99.72%	99.36%	98.66%	98.87%	98.53%
14	85.46%	90.73%	94.16%	91.64%	93.63%	94.04%	91.61%
15	86.47%	89.51%	94.87%	91.39%	93.26%	94.71%	91.70%
16	99.70%	100.00%	100.00%	100.00%	100.00%	100.00%	99.95%
17	97.28%	98.43%	98.45%	100.00%	99.74%	99.72%	98.94%
18	99.14%	99.43%	99.71%	100.00%	100.00%	100.00%	99.71%
19	98.33%	98.26%	99.08%	99.04%	98.73%	100.00%	98.91%
20	93.18%	97.45%	97.82%	97.50%	96.93%	98.21%	96.85%
21	84.50%	95.24%	92.02%	93.81%	94.36%	95.15%	92.51%
22	89.09%	96.51%	93.82%	94.67%	96.28%	97.75%	94.69%
23	89.29%	91.88%	93.09%	93.82%	92.81%	93.75%	92.44%
24	85.34%	89.97%	90.30%	92.73%	92.40%	93.57%	90.72%
25	91.79%	96.87%	96.02%	96.18%	97.81%	98.34%	96.17%
26	92.16%	96.00%	99.31%	98.20%	97.92%	98.17%	96.96%
27	93.14%	93.13%	95.93%	97.31%	96.18%	97.11%	95.47%
28	86.03%	90.96%	92.64%	94.64%	95.06%	95.58%	92.49%
29	99.68%	100.00%	100.00%	100.00%	100.00%	100.00%	99.95%
30	96.51%	99.08%	98.88%	98.18%	98.86%	98.10%	98.27%
average	93.72%	96.16%	97.02%	97.06%	97.49%	97.80%	96.54%

**Table A9:** The **accuracy** of SimID on each individual user in data splitting setting **CP**.

User	1-Shot	2-Shot	3-Shot	4-Shot	5-Shot	10-Shot	Average
21	91.14%	95.67%	95.80%	96.63%	97.08%	96.24%	95.43%
22	87.53%	91.91%	91.68%	96.07%	95.73%	96.15%	93.18%
23	83.23%	88.32%	91.20%	91.67%	92.55%	92.98%	89.99%
24	82.95%	89.46%	91.03%	92.11%	90.48%	91.60%	89.60%
25	78.31%	87.55%	89.11%	90.96%	90.56%	91.21%	87.95%
26	83.37%	90.00%	91.79%	94.36%	93.79%	95.74%	91.51%
27	86.42%	93.86%	94.89%	94.35%	95.23%	96.85%	93.60%
28	80.63%	92.70%	91.97%	92.35%	93.16%	96.49%	91.22%
29	86.90%	90.96%	91.40%	92.51%	92.60%	94.55%	91.49%
30	90.65%	93.44%	95.16%	94.83%	97.49%	99.20%	95.13%
average	85.11%	91.39%	92.40%	93.58%	93.87%	95.10%	91.91%

**Table A10:** The **precision** of SimID on each individual user in data splitting setting **CP**.

User	1-Shot	2-Shot	3-Shot	4-Shot	5-Shot	10-Shot	Average
21	85.11%	90.27%	91.61%	92.94%	93.76%	93.89%	91.26%
22	87.88%	93.53%	96.15%	95.28%	96.99%	96.15%	94.33%
23	84.79%	89.38%	90.51%	91.30%	91.04%	92.59%	89.94%
24	76.43%	85.39%	87.52%	89.22%	89.73%	93.75%	87.01%
25	88.04%	92.84%	93.95%	95.37%	96.16%	96.96%	93.88%
26	88.50%	95.98%	94.24%	95.66%	97.10%	96.87%	94.72%
27	85.08%	91.15%	92.43%	93.41%	93.01%	93.71%	91.47%
28	72.17%	82.19%	83.82%	87.22%	85.58%	90.99%	83.66%
29	95.00%	98.26%	97.48%	98.28%	98.72%	98.94%	97.78%
30	93.56%	97.23%	98.40%	98.35%	97.68%	97.82%	97.17%
average	85.66%	91.62%	92.61%	93.70%	93.98%	95.17%	92.12%

**Table A11:** The **recall** of SimID on each individual user in data splitting setting **CP**.

User	1-Shot	2-Shot	3-Shot	4-Shot	5-Shot	10-Shot	Average
21	91.14%	95.67%	95.80%	96.63%	97.08%	96.24%	95.43%
22	87.53%	91.91%	91.68%	96.07%	95.73%	96.15%	93.18%
23	83.23%	88.32%	91.20%	91.67%	92.55%	92.98%	89.99%
24	82.95%	89.46%	91.03%	92.11%	90.48%	91.60%	89.60%
25	78.31%	87.55%	89.11%	90.96%	90.56%	91.21%	87.95%
26	83.37%	90.00%	91.79%	94.36%	93.79%	95.74%	91.51%
27	86.42%	93.86%	94.89%	94.35%	95.23%	96.85%	93.60%
28	80.63%	92.70%	91.97%	92.35%	93.16%	96.49%	91.22%
29	86.90%	90.96%	91.40%	92.51%	92.60%	94.55%	91.49%
30	90.65%	93.44%	95.16%	94.83%	97.49%	99.20%	95.13%
average	85.11%	91.39%	92.40%	93.58%	93.87%	95.10%	91.91%

**Table A12:** The **F1-score** of SimID on each individual user in data splitting setting **CP**.

User	1-Shot	2-Shot	3-Shot	4-Shot	5-Shot	10-Shot	Average
21	88.02%	92.89%	93.66%	94.75%	95.39%	95.05%	93.29%
22	87.70%	92.71%	93.86%	95.67%	96.36%	96.15%	93.74%
23	84.00%	88.84%	90.86%	91.49%	91.79%	92.78%	89.96%
24	79.55%	87.38%	89.24%	90.64%	90.10%	92.66%	88.26%
25	82.89%	90.12%	91.46%	93.11%	93.28%	93.99%	90.81%
26	85.86%	92.89%	93.00%	95.00%	95.41%	96.30%	93.08%
27	85.74%	92.49%	93.64%	93.88%	94.11%	95.26%	92.52%
28	76.17%	87.13%	87.70%	89.71%	89.21%	93.66%	87.26%
29	90.77%	94.47%	94.34%	95.31%	95.56%	96.69%	94.52%
30	92.08%	95.30%	96.76%	96.56%	97.58%	98.50%	96.13%
average	85.28%	91.42%	92.45%	93.61%	93.88%	95.11%	91.96%

**Table A13:** The **accuracy** of SimID on each individual user in data splitting setting **CACP**.

User	1-Shot	2-Shot	3-Shot	4-Shot	5-Shot	10-Shot	Average
21	82.68%	86.97%	91.27%	91.65%	91.44%	91.29%	89.22%
22	84.19%	92.22%	92.48%	93.87%	93.78%	97.86%	92.40%
23	91.86%	93.28%	94.48%	95.29%	96.87%	96.79%	94.76%
24	85.51%	89.94%	93.00%	94.35%	94.73%	96.02%	92.26%
25	84.60%	90.35%	92.32%	92.25%	92.38%	94.98%	91.15%
26	78.27%	85.26%	88.61%	89.04%	91.25%	94.73%	87.86%
27	87.38%	94.28%	95.11%	96.19%	96.12%	98.05%	94.52%
28	79.08%	84.26%	89.21%	91.11%	90.63%	92.46%	87.79%
29	84.71%	91.68%	95.44%	95.33%	96.92%	98.34%	93.74%
30	94.41%	97.30%	96.18%	96.84%	97.84%	97.84%	96.74%
average	85.27%	90.55%	92.81%	93.59%	94.20%	95.83%	92.04%

**Table A14:** The **precision** of SimID on each individual user in data splitting setting **CACP**.

User	1-Shot	2-Shot	3-Shot	4-Shot	5-Shot	10-Shot	Average
21	81.87%	88.75%	89.51%	93.97%	91.79%	93.89%	89.96%
22	80.71%	86.65%	91.75%	88.27%	89.98%	90.47%	87.97%
23	85.39%	89.39%	92.43%	93.82%	94.83%	95.45%	91.89%
24	78.56%	85.05%	88.11%	89.48%	91.82%	93.20%	87.70%
25	89.81%	94.16%	94.23%	96.87%	97.26%	100.00%	95.39%
26	91.10%	93.86%	93.96%	96.13%	94.94%	96.15%	94.36%
27	82.50%	87.08%	90.67%	92.32%	91.28%	95.57%	89.90%
28	81.82%	90.19%	93.79%	91.93%	94.99%	97.15%	91.65%
29	94.39%	96.29%	97.37%	97.51%	98.22%	99.07%	97.14%
30	89.75%	95.28%	96.96%	96.65%	97.08%	98.26%	95.66%
average	85.59%	90.67%	92.88%	93.69%	94.22%	95.92%	92.16%

**Table A15:** The **recall** of SimID on each individual user in data splitting setting **CACP**.

User	1-Shot	2-Shot	3-Shot	4-Shot	5-Shot	10-Shot	Average
21	82.68%	86.97%	91.27%	91.65%	91.44%	91.29%	89.22%
22	84.19%	92.22%	92.48%	93.87%	93.78%	97.86%	92.40%
23	91.86%	93.28%	94.48%	95.29%	96.87%	96.79%	94.76%
24	85.51%	89.94%	93.00%	94.35%	94.73%	96.02%	92.26%
25	84.60%	90.35%	92.32%	92.25%	92.38%	94.98%	91.15%
26	78.27%	85.26%	88.61%	89.04%	91.25%	94.73%	87.86%
27	87.38%	94.28%	95.11%	96.19%	96.12%	98.05%	94.52%
28	79.08%	84.26%	89.21%	91.11%	90.63%	92.46%	87.79%
29	84.71%	91.68%	95.44%	95.33%	96.92%	98.34%	93.74%
30	94.41%	97.30%	96.18%	96.84%	97.84%	97.84%	96.74%
average	85.27%	90.55%	92.81%	93.59%	94.20%	95.83%	92.04%

**Table A16:** The **F1-score** of SimID on each individual user in data splitting setting **CACP**.

User	1-Shot	2-Shot	3-Shot	4-Shot	5-Shot	10-Shot	Average
21	82.27%	87.85%	90.38%	92.80%	91.62%	92.57%	89.58%
22	82.41%	89.35%	92.11%	90.98%	91.84%	94.02%	90.12%
23	88.50%	91.30%	93.44%	94.55%	95.84%	96.12%	93.29%
24	81.89%	87.43%	90.49%	91.85%	93.25%	94.59%	89.92%
25	87.13%	92.22%	93.27%	94.50%	94.76%	97.43%	93.22%
26	84.20%	89.35%	91.20%	92.45%	93.06%	95.43%	90.95%
27	84.87%	90.54%	92.84%	94.22%	93.64%	96.79%	92.15%
28	80.43%	87.13%	91.44%	91.52%	92.76%	94.75%	89.67%
29	89.29%	93.93%	96.39%	96.40%	97.57%	98.70%	95.38%
30	92.02%	96.28%	96.57%	96.74%	97.46%	98.05%	96.19%
average	85.30%	90.54%	92.81%	93.60%	94.18%	95.84%	92.05%
